# Annotations

**Published:** 1911-03-04

**Authors:** 


### ANNOTATIONS.

### The First Operation under Ether.

In the February number of the *University College Hospital Magazine*—a comparatively new monthly which bids fair to become by far the best of all our medical school journals—Dr. F. W. Cock describes that memorable occasion when Liston operated on a patient who had been anæsthetised with the then novel narcotic, ether. Dr. Cock's manner of telling the story is delightful in its brisk clearness and vivid narrative form, and we congratulate both him and the editors of the magazine on what is one of the most interesting contributions to the history of medicine in England which has recently been published. The temptation to quote from it is strong, but we refrain, for excerpts would not do justice to the manner or the matter of the article, and those who are interested in the story of anæsthesia should make a point of ordering the number and reading Dr. Cock's version for themselves. A great deal of the material is culled from old case-books, or is the transcription of notes made by eye-witnesses, while the illustrations greatly add to the interest, since some of them are unique. The patient, one Frederick Churchill, was a butler aged thirty-six, whose leg was amputated by Liston during a very short period of anæsthesia induced by William Squire with ether vapour from a peculiar inhaler. The narcosis was thoroughly satisfactory, and there can be little doubt that this was, as Dr. Cock states, the first occasion in which ether had been used in this country to induce anæsthesia for the performance of a major operation. The anæsthetic had been used before on patients who had had several teeth extracted, while its properties were, of course, fairly well known in America. Dr. Cock calls Liston's operation on Churchill "the first operation under ether in Europe," but he does not explicitly state in the course of his very interesting article on what grounds he bases this claim to priority. The whole history of anæsthetics and anæsthesia demands further investigation, and anæsthetists who take the slightest interest in their craft must be grateful to Dr. Cock, who has done so much to elucidate several points with regard to the use of anæsthetics in this country.

### The Treatment of Yaws with "606."

Dr. Richard P. Strong, in the *Philippine Journal of Science* for October 1910, records the histories of 25 cases of yaws treated by Ehrlich's dioxydiamidoarsenobenzol. Dr. Strong believes that the drug forms an ideal specific for this disease. Three or four days after its injection the granulomatous lesions begin to fade and in the course of from ten to twenty days they usually have disappeared entirely. Tumour masses measuring several centimetres in diameter and about a centimetre in thickness are absorbed in a short space of time, and under the influence of no local treatment whatever. The disappearance of such lesions and the cures produced are, to Dr. Strong's mind, nothing short of marvellous. Even where large granulomatous masses or deep ulcerations existed, these were healed within two to four weeks. A slight reddening appears about the margins of the lesions 24 to 48 hours after the injection of the preparation. The centre of the lesion then usually assumes a purplish or bluish congested appearance. Where crusts exist these frequently are not absorbed, but drop off. None of the cases treated have shown any signs of relapse, although they received but a single injection; over four months have elapsed since most of them were inoculated. In severe instances of the disease and where the ulcerations are extensive and of long standing Dr. Strong believes that a second inoculation given about three weeks after the first one may be advisable. He concludes from his observations that dioxydiamidoarsenobenzol is as important a specific for yaws as quinine is for malaria.

### A New Source of Rubber.

When in 1823 Mackintosh started a factory at Glasgow for the manufacture of the garment to which he gave his name, the world's production of rubber stood at 120 tons per annum. In 1888, when Dunlop brought out the pneumatic tyre, this production had risen to 22,000 tons a year. At the present moment more than 80,000 tons are produced yearly, and no industry in the world has shown such an example of lightning development. It is this fact which gives a particular interest to a note of M. Jean Dybowski, Professor of Colonial Agriculture at the National Agricultural Institute and at the Higher School of Agriculture, recently presented to the Paris Academy of Science relative to a new source of production of rubber. According to this note it is now possible to extract commercially from a gum called jelutong the 10 to 20 per cent. of rubber it contains. This gum is derived from the latex of a plant of the family Apocynacæe, the *Dyera Costumata* (Hooc). It is found in large quantities in the Malay States, and the gum which it produces can be obtained at a low price in the principal European markets, such as London, Antwerp, and Hamburg. The method of extraction of the rubber is a very simple and comparatively easy process, and only takes three or four hours. On the other hand, preparation costs little, and the product can be sold at a price lower than obtains in the rubber market and still yield a handsome profit. A factory which has been started has already produced more than 60,000 pounds of rubber from this source which has been sold at a low price. Moreover, other companies have used the method described by M. Dybowski, and one of them with works in Russia counts on producing this year some 400,000 pounds of rubber extracted from this gum. Since the rubber so produced is of a higher quality than that obtained from the Congo, it seems probable that, thanks to this new source, the growing demand for rubber will be met without forcing up the price of the article to the height at which it has stood during the past year.

